# Supplementary material for: Spectroscopic Investigation of DCCH and FTSC as a potential pair for Förster Resonance Energy Transfer in different solvents
Source: PLoS One. 2020 Feb 11;15(2):e0228543. doi: 10.1371/journal.pone.0228543 (PMC7012416; doi:10.1371/journal.pone.0228543)
Supplement: S1 File — (DOCX) [file pone.0228543.s001.docx]

Electronic Supplementary Information

Solubility

The solubility of the molecules in the desired solvent becomes important when the concentration of the molecules is a critical factor in the experiment. This is exactly the case with FRET as it is a highly distance dependent effect. In the end the average distance between the molecules will be determined by the concentration of the molecules in solution and the minimum distance is determined by the maximum solubility of the molecules.

In Fig 1 one can see the average distance between molecules dissolved in H2O. As an example for the calculation, we consider 10^-3^ mol of one dye molecule dissolved in 1 L of water (55.3 mol). This means that per dye molecule there are 55.3 x 10^3^ water molecules. For simplicity we assume that the water molecules are arranged in a cube around one dye particle. The average distance between two dye molecules surrounded by water cubes will then be 38 water molecules or approx. 11.8 nm. For the calculation the average distance between water molecules in bulk water was assumed to be 0.31 nm. (1) Due to molecular diffusion this average will not be a single value but a spread-out Gaussian curve. The reason why this is important is that at a certain concentration this average will be too large and the likelihood of two molecules interacting via FRET become zero as it has been described by Förster. A similar estimation has been reported by others. (2)

**
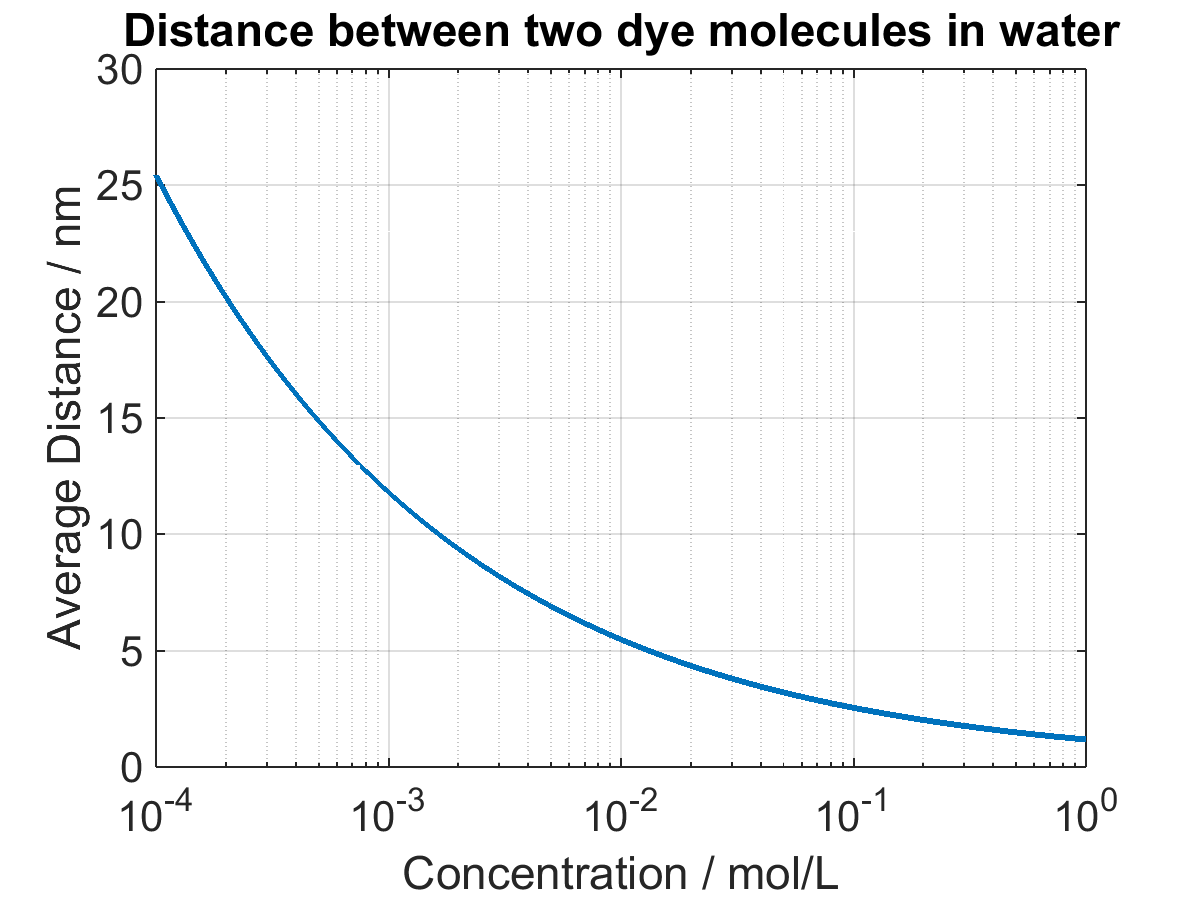
**

**Fig. S1.** Calculation of the average distance between dye molecules in solution depending on the concentration.

Additionally, in Table 1 the maximum solubilites of the dyes in the used solvents were determined. The dyes showed good solubility except for H2O. Therefore, in H2O saturated solutions were produced for DCCH and FTSC using a) 147 mg/L and b) 85 mg/L.

Additionally, in Table 1 the maximum solubilites of the dyes in the used solvents were determined. The dyes showed good solubility except for H_2_O.

**Table S1.** Solubility limit of Molecules in different solvents in mmol/L.

| Solvent  Molecule | H_2_O | DMF | DMSO | THF |
| --- | --- | --- | --- | --- |
| DCCH | 0.5 ± 0.1 | 35 ± 2.4 | 17.1 ± 0.9 | 20 ± 1.1 |
| FTSC | 0.2 ± 0.02 | 26.8 ± 2.0 | 12.4 ± 0.7 | 5.8 ± 0.3 |

pH

As described in the experimental section the pH of the solutions was adjusted by Triethylamine to investigate its effect on the spectroscopic properties of the fluorescent molecules. Table S2 shows how much effect in terms of pH the mentioned volumes had on the solutions.

**Table S2.** Measured pH and conductivity of the used solvents under neutral and alkaline conditions.

|  | pH Neutral / - | pH alkaline / - | Conductivity (neutral) / mV | Conductivity (alkaline) / mV |
| --- | --- | --- | --- | --- |
| H_2_O | 8.5 ± 0.2 | 11.8 ± 0.2 | -89 ± 2 | -238 ± 4 |
| DMF | 12.4 ± 0.1 | 12.5 ± 0.1 | -321 ± 5 | -327 ± 5 |
| DMSO | 10.9 ± 0.1 | 11.4 ± 0.2 | -230 ± 5 | -260 ± 5 |
| THF | 6.6 ± 0.1 | 10.2 ± 0.1 | 16 ± 1 | - 181 ± 3 |

FRET Efficiency

The FRET efficiencies of the dyes in solution was calculated for acceptor sensitation by Equation (4) and for donor quenching using the following equation.

$$\eta_{DQ}=1-\frac{I_{DA}}{I_{D}}$$

Where ηDQ [-] is the FRET efficiency from donor quenching. I_DA_ and I_D_ are the intensities of the Donor in the presence and absence of the acceptor, respectively The used parameters and calculated results can be found in Table S3.

**Table S3**. Parameters, measured intensity values and calculated FRET efficiencies for the 5 working systems.

| System | Acceptor | Excitation | ε DCCH [M^-1^cm^-1^]) | ε FTSC [M^-1^cm^-1^]) | I_A_ [a.u.] | I_AD_ [a.u.] | I_D_ [a.u.] | I_DA_ [a.u.] | AS [-] | DQ [-] |
| --- | --- | --- | --- | --- | --- | --- | --- | --- | --- | --- |
| 0.1mM DMF | DCCH | 330 nm | 0,1289 | 0,2271 | 926743 | 2,12E+06 | 1,04E+07 | 114421 | 0,73 | 9,89E-01 |
| 0.1mM DMSO | DCCH | 330 nm | 0,0253 | 0,2538 | 696170 | 1,72E+06 | 6,05E+06 | 170012 | 0,14 | 9,72E-01 |
| 1 mM DMSO | DCCH | 330 nm | 0,0253 | 0,2538 | 1484 | 4723 | 7813 | 0 | 0,22 | 1,00E+00 |
| 1 mM THF | DCCH | 335 nm | 0,0207 | 0,2163 | 12361 | 24494 | 17112 | 0 | 0,094 | 1,00E+00 |
| 0.15 mM H2O | FTSC | 420 nm | 2,129 | 0,1392 | 22413 | 34198 | 37474 | 12465 | 0,034 | 6,67E-01 |
| 1 mM DMF (alkaline) | FTSC | 420 nm | 5,257 | 0,1635 | 8034 | 12798 | 3249 | 1794 | 0,018 | 4,48E-01 |
