# Supplementary material for: Spectroscopic Investigation of DCCH and FTSC as a potential pair for Förster Resonance Energy Transfer in different solvents
Source: PLoS One. 2020 Feb 11;15(2):e0228543. doi: 10.1371/journal.pone.0228543 (PMC7012416; doi:10.1371/journal.pone.0228543)
Supplement: Attachment — Submitted filename: Response to Reviewers.docx [file pone.0228543.s002.docx]

The authors thank both reviewers for their helpful comments. We have revised the manuscript and believe that it has led to a significant improvement of the work. Since the labelling of the Figures and Tables has changed we uploaded the new Figures as well.

Reviewers' comments:

Reviewer's Responses to Questions

***Comments to the Author***

1. Is the manuscript technically sound, and do the data support the conclusions?

The manuscript must describe a technically sound piece of scientific research with data that supports the conclusions. Experiments must have been conducted rigorously, with appropriate controls, replication, and sample sizes. The conclusions must be drawn appropriately based on the data presented.

Reviewer #1: Yes

Reviewer #2: No

------------------------------------------------------------------------


2. Has the statistical analysis been performed appropriately and rigorously?

Reviewer #1: N/A

Reviewer #2: No

------------------------------------------------------------------------


3. Have the authors made all data underlying the findings in their manuscript fully available?

The PLOS Data policy [<http://www.plosone.org/static/policies.action#sharing>](http://www.plosone.org/static/policies.action#sharing)requires authors to make all data underlying the findings described in their manuscript fully available without restriction, with rare exception (please refer to the Data Availability Statement in the manuscript PDF file). The data should be provided as part of the manuscript or its supporting information, or deposited to a public repository. For example, in addition to summary statistics, the data points behind means, medians and variance measures should be available. If there are restrictions on publicly sharing data—e.g. participant privacy or use of data from a third party—those must be specified.

Reviewer #1: Yes

Reviewer #2: No

------------------------------------------------------------------------


4. Is the manuscript presented in an intelligible fashion and written in standard English?

PLOS ONE does not copyedit accepted manuscripts, so the language in submitted articles must be clear, correct, and unambiguous. Any typographical or grammatical errors should be corrected at revision, so please note any specific errors here.

Reviewer #1: No

Reviewer #2: No

------------------------------------------------------------------------


5. Review Comments to the Author

Please use the space provided to explain your answers to the questions above. You may also include additional comments for the author, including concerns about dual publication, research ethics, or publication ethics. (Please upload your review as an attachment if it exceeds 20,000 characters)

Reviewer #1:

Dear Reviewer #1:

Thank you for the helpful comments. We tried to assess them as good as possible.

The MS entitled, "Spectroscopic Investigation of DCCH and FTSC as a potential pair for Förster Resonance Energy Transfer in different solvents" by Ulrich Hirn et al., presents experimental evaluation of two molecules FTSC and DCCH in various solvent as function of varying pH and the results based on steady state absorption and fluorescence spectroscopic measurements.
The Introduction section is quite long and needs to be trimmed. The paragraph (page 3; lines #81-86) may appropriately be shifted to the end of Introduction section.

The introduction was slightly shortened and the paragraph was shifted to the end of the introduction.
In eqn 5, the attenuation coefficient epsilon is not described in the following text, as it appears for the first time.

Epsilon was introduced in the text
In Results, on page 8; lines # 232-235, the sentence needs to be revised as it is unable to provide correct meaning.

Sentence was revised
Similarly, on page 9; lines 281-283, the sentence be revised to give the reader correct meaning.

Sentence was revised
Table 2 on page 10, should mention whether the values provided are absolute or % values.

We indicated in the header of the table that the QY is dimensionless.
On the same page, lines # 313-314 the sentence "Another important ...... spectra of molecules" is incorrect both grammatically as well as scientifically. Rewrite it.

We revised the sentence grammatically but were unsure about the scientific point.
On page 10, paragraph (lines #407-412), should consider the contribution of radiative transfer as the distance of approx. 16.4 nm (quite greater than 2xForster distance) is also able to show FRET signal.

It is generally assumed that in the range of ½ R_0_ – 2 R_0_ the predominant transfer mechanism is FRET and the other mechanisms can be neglected. Reabsorption apparently takes place longer than 1000 nm which is far beyond our distances. In Table 4, the authors may give the values of epsilon, QY, R0 instead of giving symbols to show their strengths.

The Table was redone and holds quantitative values now. The authors agree that the addition of the numerical values is a clear improvement of the work.

Please clarify whether, the intensity values mentioned in Fig. 7 are in rel unite or counts.
While the results are interesting, the discussion of the same needs to be presented with more lucidity.

All the Figures containing Intensity scales were redone to increase the clarity. As the units on the y axes are in this case not that important we decided to label them as arbitrary units (a.u.).

The quality of the paper would greatly enhance if the authors include the time resolved measurements (fluorescence lifetimes) also which confirm the resonance energy transfer process in such mixtures.

The authors are aware that Fluorescence Lifetime measurements are an excellent and very compelling way of measuring FRET. However, we do not have access to such an equipment. A few lines were added in the Results and Discussion section which address this issue.

The authors are advised to take the assistance of native English speaker to improve the language of the MS. As it is, there are several grammatical errors, punctuation errors and incorrect sentences leading to unclear information. H2O is mentioned at several places without proper use of subscript. Whole MS may be checked for such mistakes. The paper may be accepted after appropriately revising it.

We changed all H2O to H_2_O. The English was revised by our institutions English checking service..

We want to thank Reviewer #2 for his/her highly helpful comments. Although the revision was very tough the comments were written in a very respectful and inteligible manner which the authors appreciated very much. We actually believe that every comment helped to improve the manuscript strongly.

Reviewer #2: The manuscript describes the spectroscopic analysis of two dyes in a variety of solvents that have been used as a FRET pair in a previous publication to measure the contact between paper fibers. The manuscript focuses mostly on the spectroscopic properties of the molecules in solvents. Significant issues with the manuscript exist that reduce its clarity, reproducibility, and impact as enumerated below.
1. FRET requires the molecules to be close together to give signal so typically molecules in solution are not used since they are far apart. The manuscript uses an uncommon method to determine the FRET parameters for these molecules and thus other peer reviewed papers should be referenced to support the method they use to perform FRET in solution. This would strengthen their argument that this is a valid method for FRET measurements.

The method used for the determination of the Förster distance was taken from Chapter 5 of the book FRET – Förster Resonance Energy Transfer by Igor Medintz and Niko Hildebrandt. The method appears to be indeed a valid one. A quick literature research also supports this.

Kremers, Gert-Jan, Joachim Goedhart, Erik B. van Munster, and Theodorus W. J. Gadella. “Cyan and Yellow Super Fluorescent Proteins with Improved Brightness, Protein Folding, and FRET Förster Radius,.” *Biochemistry* 45, no. 21 (May 1, 2006): 6570–80. <https://doi.org/10.1021/bi0516273>.

2. Another curious statement on page 4, line 124, is that the authors were “satisfied with the qualitative result” for the transfer efficiency. The authors should justify why they do not wish to quantify the FRET efficiency.

We agree that the original text passage in the manuscript was quite strange. In the revised manuscript we are reporting and discussing the FRET efficiencies in the results section.

3. The authors should include a paragraph at the end of the introduction to summarize the goals, hypotheses, and motivations for the work they did as these are not clear.

This comment matches a comment from referee#1 and was edited by changing the position of a paragraph in the introduction section to the end of the section and rewriting the paragraph slightly.

4. The method to determine solubility appeared to be non-standard. Typically, a shake flask method should be used to determine solubility by using a fixed time for sample to dissolve. Specific criteria were not given to determine when a sample was considered fully dissolved or not.

The authors agree that the solubility is really not such a relevant property here. Also it was written in a confusing manner. Therefor it was cancelled from the manuscript and we will report it in the supplementary information as it does hold some value since the solubility might be a limiting factor for some purposes.

5. On page 6, line 167, the authors state that the same amount of triethylamine was added to each solution “to introduce the same amount of OH- ions to their system.” The only solvent that is protic is water. All others would not produce hydroxide ions. This phrase should be changed.

The authors agree here that this was expressed strangely. We have rewritten the section.

6. On page 6, line 169, 10-times the triethylamine concentration was used in THF because otherwise it “did not yield any effect.” What effect is not explained in the methods section here and it should be. Why not use this same concentration for the other solvents?

The section was rewritten

7. Line 191 page 6, pHema is not defined.

Poly Hema was defined in the article.

8. The preparation of samples in pHema and on paper fibers was referenced to their previous paper, ref 22. The authors should be clear in this paper the concentration of dye on fibers and in films and how this was determined.

The concentration of dyes in the films can be easily determined by using the amount of dye and the volume of pHema. However, for the underlying investigations this was not necessary.

For the fibers we used an approximantion to evaluate the concentration. With the measured quantum yield and the assumption that the molar attenuation coefficient is not significantly different than the one of the dyes in pHema the concentration on the fibers were calculated by comparing the intensity values of recorded images using fluorescence microscopy.

9. The authors should justify with literature the calculation they used to determine the distance between dye molecules. A sample calculation should be provided.

The calculation of the average distance was put into the ESI along with a citation that an estimation such as this one was already performed by others.

10. The Table 1 data are not consistent with the description of the method. The method made is sound like all solutions were saturated, but the presented results suggest that only water was saturated. If this is the case, the method should be changed and the title of the table should be changed to concentration in solution as it is not the solubility limit. Also, the concentration of the dyes in water should be given in mmol/L to be consistent with the table results.

The term solubility was changed to the hopefully more clear term solubility limit and the quantities are now all reported in mmol/L. As mentioned above the Table can now be found in the ESI.

11. On line 239, the authors stated that they used the same amount of alkalinity by adding the same amounts of triethylamine (TEA) to the solvents. Since the solvents have different polarities, this statement is not true since the pKb of TEA will change. The discussion around the TEA addition is weakened by not knowing where the equilibrium of FTSC lies in terms of towards it absorbent or non-absorbent state.

We have increased the alkalinity in all solvents using TEA. Althought that the change in pH was often very small the effect it had on the spectra was significant. The final pH values are now given in the ESI.

12. The authors mention a NaOH reference on line 255, but do not state what this in reference to as it is not described in the methods.

In the materials section there is a paragraph that explains what the references are. However, for clarity it was reiterated in the label of the figures and in the legends.

13. The authors mention a chemical modification to the hydrazide group of the DCCH but they do not describe how this was done in the methods. For example, purification would affect the final pH of the mixture and the fluorescence behavior.

The authors agree that an explanation of the procedure is missing in the M&M section. Therefore, the chemical modification was described in the Materials and Method section. From Table 1 one can see that the QY of DCCH is not strongly affected by a changing pH. However after the modification a significant increase of the QY at the same pH as for the unmodified molecule in THF can be observed.

14. Since the method of pHema films with fluorescent dyes was not clear, it is not clear whether the concentration of the dyes are the same. This makes it difficult to assess the claim on page 9 line 292 that the QY increased as much as they state it did.

In theory the QY should be independent of the concentration. However, we are aware that deviations can occur especially due to the inner filter effect already at low concentrations. Thus we have added the important statement that the QY of the materials were performed by making sure that the measured absorbance is at or below approx.. 0.05 ODs at λ_max_. At this low ODs the influence of the inner filter effect can be neglected. This is also according to literature.

15. The QY measurement error seems high. What is the origin of the error? Is there an experimental method issue?

After consulting the literature we were a little disappointed as many authors do not report errors on the QY measurements at all. Our 20 % error stems from statistics. Additionally, we used the gaussian error propagation method to propagate realistic errors on the relative QY formula:

1. Repeated measurements gave an error between 10 and 20 % for the different samples.
2. Gaussian error propagation method yielded an error of 10 %.

- We have chosen to report an error of 20 % as it is the more pessimistic value.

16. The sentence on line 436, “Due to this investigation… dyed with DCCH and FTSC.” Is confusing. The reference paper has already been published so the work of the current paper does not seem to have enabled that work. Also, all the current work was in solution not a pHema material.

In order to better describe the time sequence of the work carried out we have revised the manuscript according to the suggestion of the reviewer: *“Due to this investigation it was possible to develop an already published method to investigate interactions between surfaces by measuring the adhesion between pHema thin films dyed with DCCH and FTSC. (21)“*

17. In Table 4 what was the criteria for high or strong (low or weak) FRET signal? This analysis seems very qualitative.

We agree with the reviewer. We changed the Table and are now reporting quantitative values. This way our conclusions become more clear.
18. The authors did not clearly discuss how solution based FRET can be translated to surface-surface interaction FRET.

“These investigations of the FRET systems in solution were later adapted to measure adhesion between pHEMA films. (21). Cellulosic surfaces, however, are developing full adhesion only when they are in contact during drying from the water swollen state(27). The investigated system therefore is, due to the instability of its fluorescence characteristics in water, only partly suited to study the adhesion between cellulosic surfaces.”

[27] Hirn U, Schennach R. Fiber-fiber bond formation and failure: mechanisms and analytical techniques. In: Proceedings of the 16th fundamental research symposium, Oxford. 2017. p. 839–64.
